# Tennis Timing Assessment by a Machine Learning-Based Acoustic Detection System: A Pilot Study

**DOI:** 10.3390/jfmk10010047

**Published:** 2025-01-27

**Authors:** Lucio Caprioli, Amani Najlaoui, Francesca Campoli, Aatheethyaa Dhanasekaran, Saeid Edriss, Cristian Romagnoli, Andrea Zanela, Elvira Padua, Vincenzo Bonaiuto, Giuseppe Annino

**Affiliations:** 1 Sports Engineering Laboratory, Department of Industrial Engineering, University of Rome Tor Vergata, 00133 Rome, Italy; lucio.caprioli@uniroma2.it (L.C.); ameni.najlaoui@ept.ucar.tn (A.N.); francesca.campoli@uniroma5.it (F.C.); aatheethya2703@gmail.com (A.D.); saeid.edriss@alumni.uniroma2.eu (S.E.); vincenzo.bonaiuto@uniroma2.it (V.B.); 2 Department of Human Science and Promotion of Quality of Life, San Raffaele Rome University, 00166 Rome, Italy; cristian.romagnoli@uniroma5.it; 3 Robotics and Artificial Intelligence Laboratory—ENEA “Casaccia” Research Centre, 00123 Rome, Italy; andrea.zanela@enea.it; 4 Human Performance Laboratory, Centre of Space Bio-Medicine, Department of Medicine Systems, University of Rome Tor Vergata, 00133 Rome, Italy; giuseppe.annino@uniroma2.it

**Keywords:** tennis, timing, acoustic detection, machine learning, performance analysis

## Abstract

**Background/Objectives:** In tennis, timing plays a crucial factor as it influences the technique and effectiveness of strokes and, therefore, matches results. However, traditional technical evaluation methods rely on subjective observations or video motion-tracking technology, mainly focusing on spatial components. This study evaluated the reliability of an acoustic detection system in analyzing key temporal elements of the game, such as the rally rhythm and timing of strokes. **Methods:** Based on a machine learning algorithm, the proposed acoustic detection system classifies the sound of the ball’s impact on the racket and the ground to measure the time between them and give immediate feedback to the player. We performed trials with expert and amateur players in controlled settings. **Results:** The ML algorithm showed a detection accuracy higher than 95%, while the average accuracy of the whole system that was applied on-court was 85%. Moreover, this system has proven effective in evaluating the technical skills of a group of players on the court and highlighting their areas for improvement, showing significant potential for practical applications in player training and performance analysis. **Conclusions:** Quantitatively assessing timing offers a new perspective for coaches and players to improve performance and technique, providing objective data to set training regimens and optimize game strategies.

## 1. Introduction

Tennis is considered a coordinative sport, and the technique adapted to the tactical context plays a key role in the success of the motor task [1]. Unlike sports in which the outcome depends solely on the athlete’s ability and talent, during the match, the tennis player is subjected to tactical conditioning and the opponent’s countermoves, being faced with always different situations in terms of placements, ball speed, and spin (i.e., Open-Skill situations) [2]. The goal is to play in such a way that the opponent can no longer reach the ball or lead him into error [3,4]. To this purpose, the player has to act by taking away time or space from the opponent, using various coordination skills such as reaction, transformation, balance, spatial–temporal orientation, movement combination, and rhythmization [5,6]. Indeed, the coordination and synchrony of the various body segments allow the athlete to play powerful and accurate strokes [7,8], and it is achieved through the sequential activation of muscle groups, coordinated torso rotation, and execution with proper timings of ball impact [9,10]. Hence, the kinetic chain principle clarifies how energy is transferred sequentially from the lower limbs through the trunk and arm during a tennis stroke, showing the importance of harmonizing movement patterns for optimal performance [11,12,13]. Recent studies have highlighted, among others, how temporal harmonic structures can be identified in tennis players of good playing ability, emphasizing the attention needed to the temporal components of technical stroke execution [14,15].

Since the ability to have rhythm and combine movements is crucial in the quality of the shot [1,7,8], the measure of some temporal components of the game, such as rally rhythm and groundstroke execution timing (defined below), can be a helpful tool for evaluating players’ technical–tactical skills and, at the same time, identify a training aid.

The rally’s rhythm refers to the frequency of strokes played and is expressed in shots per minute [16]. It depends on the speed, effect, and trajectory of the ball, anticipation, and the position of the two players on the court, and it is related to the level of play. Indeed, players with more experience and technical skills can play a rally at a higher rhythm without errors [16,17,18]. In addition to this skill, expert players also know how to vary the rhythm of the rally, which represents an essential tactical skill that allows them to disrupt the opponent’s play and can be learned and trained in practice [17,18]. For this reason, the measure of this parameter is often used as a technical test for on-court evaluation. (A typical rally test consists of counting the number of valid shots played in a one-minute rally from the baseline. Players must have continuity in the rally, and the coach should provide them with the ball immediately when the rally stops. Professional players can play an average of 42–48 shots per minute [19]). In this context, BPM (Beat Per Minute) applications (BPM apps generally have a button to be pressed by the operator at each shot played and can calculate the average frequency and the instantaneous frequency derived from each individual rally (between two shots)) are currently used to measure rally frequency (e.g., [20]). However, this manual technique (like a stop-watch) could present considerable operator–dependent errors [21,22].

In the technical analysis of rebound shots, great attention is generally paid by coaches to the measurement of the spatial components of the gesture, such as lengths and angles [23,24,25,26], and it is not usual to consider the temporal analysis of the movement, which is instead an essential part [2,15]. The term timing is used in the literature, referring to the harmonious sequence of trunk, extremity, and racket movements that allow the ball’s trajectory to be intercepted at the optimal instant and thus with maximum effectiveness [19]. The temporal sequence of body movements and racket trajectories is adapted according to the type of shot played and the tactical situation, contributing to stroke efficiency and consistency [8]. This adaptation may also occur among different playing surfaces, although it was not found statistically significant [27]. The executive timing of rebound shots in tennis (groundstrokes) can be identified by measuring the time between the ball rebound and the impact on the string pattern. Proper timing (neither too short nor too long) can affect performance, and it can allow the ball to be impacted at a sufficient distance from the body in an optimal spot, promoting greater stroke effectiveness and less strain on the muscle–tendon and joint structures, which consequently reduces the risk of injury [28,29].

Despite its critical importance, the assessment of executive timing in tennis remains complex and multifaceted, but it cannot be ignored. Among the most currently used methods to measure the spatial–temporal components of the game are radar detection systems [30], wearable sensors [31,32], or 2D and 3D video motion-tracking systems [2,33,34,35,36,37].

Since, in tennis, the game takes place without environmental and audience disturbances (e.g., the chair umpire provides for calling out the rowdy audience before the start of the point of having a point replayed if it is disturbed), the sounds that characterize the game are well recognizable, and therefore, the use of acoustic assessment systems can be of interest for their potential and ease of use. Some studies have used acoustic analyses of the game to detect the ball’s impact with racket strings or rebound on the ground, as well as spin effects [38,39,40,41]. Some of these studies adopted peak energy detection and frequency analysis approaches [39]. In another study [42], the authors proposed a combination of video and audio analysis techniques to study the ball’s trajectories. In this field, some authors proposed an acoustic methodology for on-court timing assessments [43].

These approaches involved an analysis, mainly for research purposes, which included conducting a posteriori. However, although the studies mentioned above were not focused on direct application in the field due to more elaborate processing, they, both those based on audio systems and video motion-tracking techniques, prove to be valuable analysis tools and may be useful as a reference for a faster and more automated system.

The present study aims to validate an ML-based acoustic detection algorithm to assess rallies’ rhythms and groundstrokes’ executive timings and give immediate feedback. We hypothesize that the proposed algorithm may be able to analyze game timing in a few seconds with adequate accuracy from audio recordings made by commercial microphones, regardless of the type of racquet, ball, or string tension used. The developed system is proposed to overcome the limitations of previous studies and carry out immediate feedback that is a useful on-court application for players’ assessment and training. In this regard, after the validation of the system, we will present an example of a possible on-court application to evaluate the rally rhythm of four players and the timing of rebound shots of a heterogeneous group of six amateurs (this is not intended to be a statistical investigation). As described in the following paragraphs, interesting data may emerge for training to understand the level of automation of the technical gesture and experience level.

## 2. Materials and Methods

Two data-acquisition protocols were conducted for this study: one focused on analyzing the rally rhythm and the other on the executive timing of groundstrokes.

### 2.1. Data Collection

A total of ten healthy subjects participated in the study (three females and seven males), of which four male subjects were involved in the rally assessment test and a further group of six subjects (three females and three males) in the groundstrokes assessment test. All measurements were conducted in outdoor courts with different environmental conditions. The Internal Research Board of the “Tor Vergata” University of Rome approved the study. All the procedures involved in this study were in accordance with the Declaration of Helsinki.

#### 2.1.1. Rally Assessment

**Figure 1:**
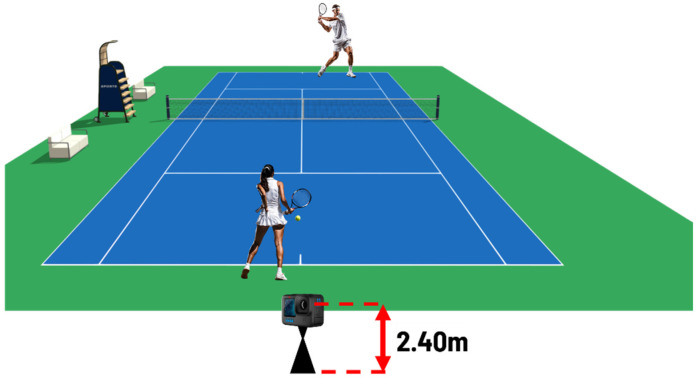
Schematic representation of the camera placement (on the back fence of the court, 2.4 m above the ground) during rally measurements protocol.

Four healthy male subjects (21.1 ± 3.7 years) ranked in the second category by the FITP (Italian Tennis and Padel Federation) were recruited for the rally assessment protocol. The subjects were asked to play a baseline rally at an average rhythm. An action camera (GoPro 11 by GoPro Inc., San Mateo, CA, USA) was placed on the back fence of the court (under the test player’s side) at a height of 2.4 m above the ground (Figure 1).

The camera-shooting mode was set at 240 fps and 1920 × 1080 resolution with stereo audio recording from the built-in microphone set at 48 kHz. The camera position was chosen for the practicality of recording the entire rally of the two players and its low invasiveness, as there are no tripods in the playing area. Two subjects (2.8 FITP rank) performed the rally on a hard surface on a windy day (the wind speed was 24 km/h according to measurements from the Rome Ciampino meteorological station) with sound disturbances coming from the adjacent court (Rally 1), and the other two subjects (2.4 FITP rank) did so on a day with a light wind (the wind speed did not exceed 15 km/h according to measurements from the Rome Ciampino meteorological station) on a clay court with sounds coming from an adjacent court (Rally 2).

#### 2.1.2. Groundstrokes Assessment

**Figure 2:**
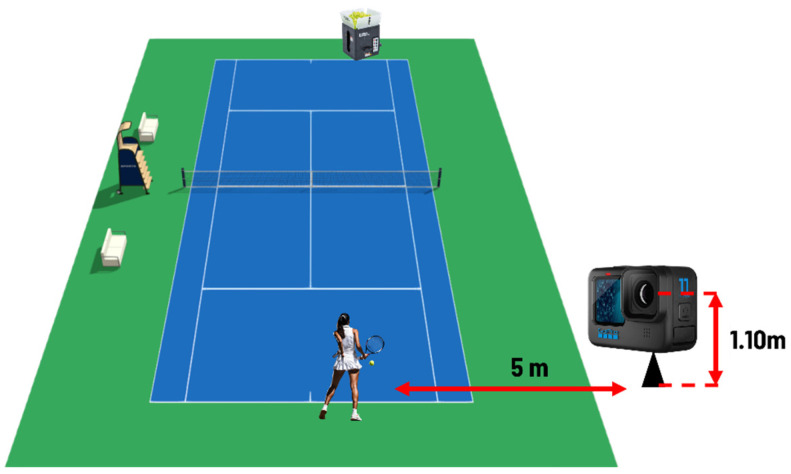
Schematic representation of the camera placement (5 m distance laterally to the player 1.10 m above the ground) and ball-throwing machine during ground stroke protocol measurements.

Six healthy subjects (three females and three males, 40.8 ± 13.6 years) of different playing levels were recruited for the groundstrokes assessment and divided by playing level into two subgroups: G1 (n = 4) and G2 (n = 2). In G1, four participants (three females and one male, 49.8 ± 3.6 years) had an average of 1.8 years of experience; in G2, two participants (two men, 23.0 ± 7.0 years) had an average of 3.3 years of experience. The acquisitions were made in two days after 20 min of physical and technical warmups. The two groups played forehand shots on a clay court, receiving an easy-to-handle ball from the coach, totaling 128 shots (80 in G1 and 48 in G2). Environmental noises were present in both situations, including wind, voices, and road traffic in the former case and sounds from adjacent fields in the latter. An action camera (GoPro 11 by GoPro Inc., USA) was placed at a 5 m distance laterally to the player 1.10 m above the ground and set to shooting mode at 1920 × 1080 at a sampling rate of 240 fps with stereo audio recording from the built-in microphone set at 48 kHz (Figure 2). This camera position was chosen to capture the entire technical gesture and bounce of the ball at a height corresponding closely to the impact point and to record both sounds (rebound and impact) correctly.

### 2.2. Peaks Detection

**Figure 3:**
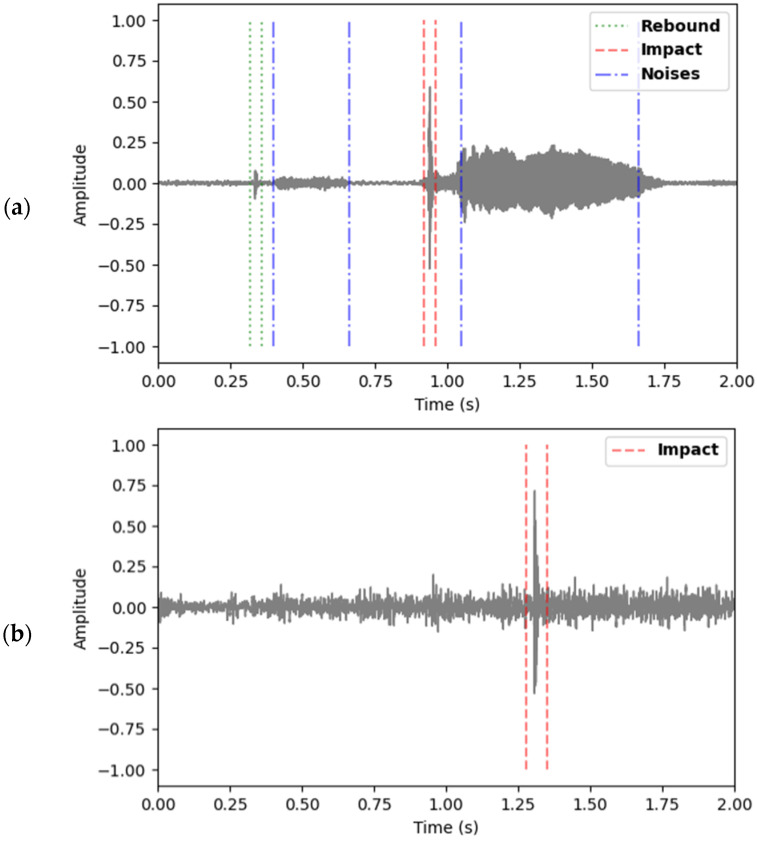
Waveforms of the sound signal are shown in linear view with normalized amplitude values (the normalization of the amplitude according to Resource Interchange File Format (RIFF) specifications, typical of the wav file format, where the value −1 represents the minimum amplitude, the value 1 represents the maximum amplitude, and 0 represents silence [44]): dashed lines indicate the shot impact; dotted lines indicate the rebound; dash–dot indicate noises (shoes and voice). In (**a**) background noise is minor, whereas in (**b**) it is more prominent.

The audio signal recorded by the camera was extracted from the video and processed by properly developed software for filtering and peak identification. The audio recordings of a match or practice session can include several types of sounds, for example wind, voices, and noises from the stands or other courts. Figure 3a depicts a waveform where the sounds of the ball impacting with the strings (“*impact*”) and the ground rebound (“*rebound*”) appear distinguishable from other sounds, such as voices and background noises.

The *impact* sound is characterized by a sharp, brief burst of high-frequency energy due to the intense, rapid vibration of the strings, while the *rebound* has a lower-frequency signature, resulting in a deeper tone. On the contrary, background noise, such as speaking or yelling, shows irregular and diverse patterns throughout the frequency spectrum, lacking the structured and predictable nature of *impact* and *rebound* sounds. However, in some cases, the events do not appear so easily recognizable, particularly the sound of *rebound*, which may be masked by ambient noises such as wind, as shown in Figure 3b. Furthermore, one may face different sound characteristics depending on the type of shot played (spin, for example) and the playing surface.

**Figure 4:**
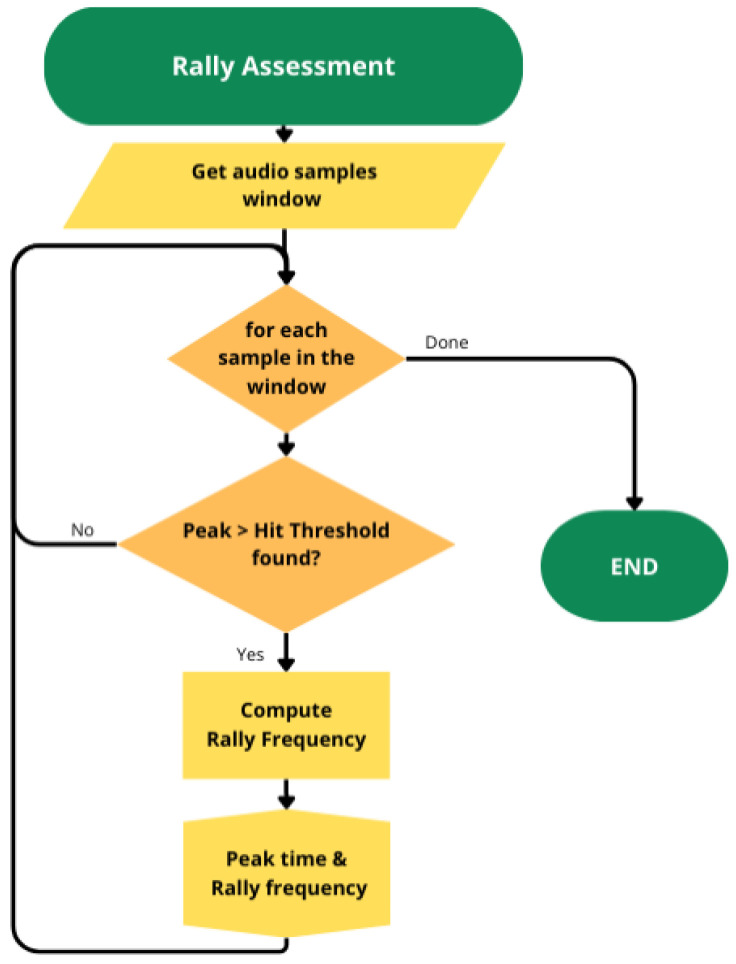
Rally assessment algorithm flow charts showing the peak detection process, corresponding to the closer player *impacts* and rally frequency calculation.

For the rally assessment, signal peaks exceeding a normalized amplitude of 0.4 and with a minimum interval between them of 390 ms were detected. The normalized amplitude value was selected based on the analysis of all processed signals, and the time interval was arbitrarily chosen since it was conservatively assumed that two impacts cannot occur in a time frame of less than 0.4 s in a baseline rally. Position, peak amplitude, and instantaneous rally frequencies, computed from the time between two consecutive *impacts* by the same player, were exported (Figure 4).

**Figure 5:**
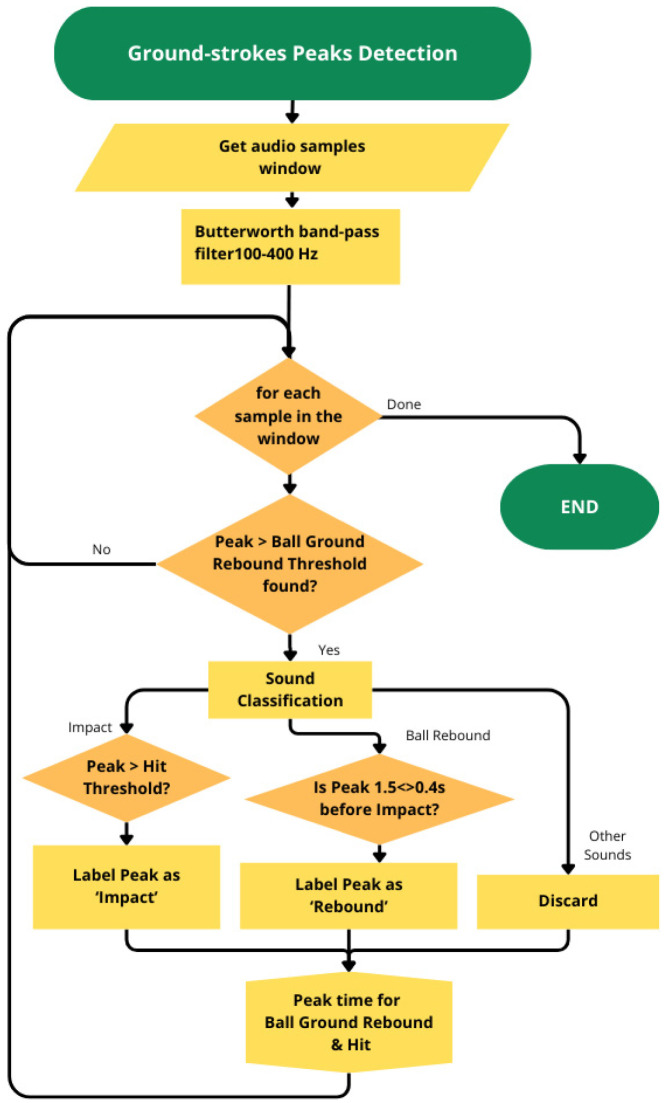
Groundstrokes peaks detection algorithm flowcharts. A Butterworth low and high pass was applied, and each peak was classified as possible *impact*, *rebound*, or noise.

On the contrary, a preprocessing stage was required before the identification task for the groundstroke peak detection because the ball-bouncing sound is often lower in amplitude than the environmental noise. So, frequency filtering was applied to denoise the signal by a five-order Butterworth band-pass filter with 100–400 Hz cut-off frequencies [45,46]. All peaks with a normalized amplitude exceeding 0.09 and peaks greater than an amplitude of 0.02 with a minimum interval between them of 100 ms were detected (Figure 5).

The normalized amplitude values were chosen based on the analysis of all processed signals, and the time interval was arbitrarily chosen since, in the structured situation analyzed, it cannot be lower than 100 ms. Peaks with amplitude exceeding 0.09 were labeled as *impact*, and those present in a time window of 1.5 s to 0.4 s before the *impact* was labeled as *rebound*.

The analysis of the audio signal for the identification of peaks related to the *rebounds* of the ball on the court or its *impact* on the racquet string plate, carried out using amplitude evaluation alone, although appropriately filtered and windowed, returns spurious results in output that require appropriate procedures for their removal.

These procedures are particularly needed in the second case (i.e., in analyzing the timing of groundstrokes), where the previously described algorithm returns more than one result for the *rebound*, and subsequent classification is required for the true *rebound* identification. In this instance, the filtering process, reducing the total number of audio events that the neural network must classify, effectively reduces the computational load.

### 2.3. Machine Learning Sound Classification

The machine learning model can precisely categorize each peak as either a noise event or a tennis ball hit by examining the sound features corresponding to each peak. So, to achieve this categorization, a machine learning approach designed specifically for sound classification was employed [47,48,49,50,51]. The model was trained to recognize and differentiate the sound of a tennis ball from other noises commonly present in a tennis court environment.

The algorithm was trained based on a dataset containing 1400 audio samples previously acquired and is described in the following paragraphs.

#### 2.3.1. Data Collection and Feature Extraction

Several sound events were collected from tennis game actions to prepare the dataset, paying particular attention to the unique noises produced when the ball hits the racket strings (*impact*) and bounces off the ground (*rebound*); we gathered 509 sounds for the class noise, 442 for *rebound*, and 448 for the *impact*.

The features used for the classification models are extracted from the audio data using a variety of time-domain and frequency-domain characteristics. First, the Short-Time Fourier Transform (STFT) is applied to compute the spectrogram, which is generated using a window size of 320 samples with a Hann window applied to reduce spectral leakage, and each window includes zero padding. The mean, standard deviation, and maximum magnitude are derived from the spectrogram for each frequency bin.

The frequency at which the maximum energy occurs is also recorded. Further, the zero-crossing rate (ZCR), spectral centroid, and Mel-frequency cepstral coefficients (MFCCs) (MFCC is preferred over cepstral coefficients in this kind of application because it focuses on meaningful features, it is more robust to noise and ensures better performance in real-world scenarios, such as tennis matches, where background noise is prevalent, making the audio classification tasks particularly effective) are extracted to capture more detailed spectral characteristics of the audio [52].

These features are then augmented with statistical properties of the audio signal in the time domain, such as the overall mean, standard deviation, and specific windowed metrics (e.g., minimum, maximum, mean, and standard deviation across short time segments). This combination of spectral and time-domain features provides a comprehensive audio representation, allowing the ML model to isolate the *impact* events from the noise.

The same strategy did not allow a second feature to isolate the *rebounds* from the noise. Therefore, further features were added to the model to improve its performance. These included tonnetz characteristics (which describe harmonic characteristics), spectral contrast (which quantifies the amplitude difference between the highest and lowest points in a sound spectrum), and chroma features (which capture pitch information).

In particular, the most informative features were chosen by the SelectKBest technique with the ANOVA F-test to reduce overfitting and increase model efficiency, focusing on those that showed the strongest correlation with the target labels.

**Figure 6:**
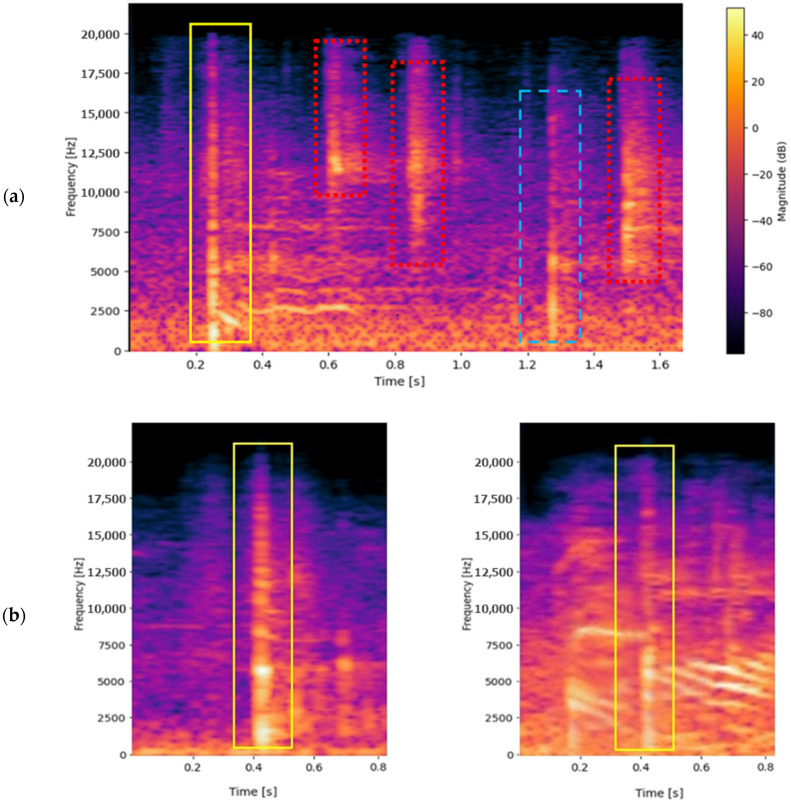
Spectrogram of an audio segment (**a**) with a ball *impact* (yellow solid line), a ball *rebound* (blue dashed line), and background noises (red dots); two different spectrograms (**b**) with a ball *impact* (yellow solid line) distinguishable on the left or within a noise on the right.

Figure 6a shows an example spectrogram of an audio segment with a ball *impact*, background noise, and a ball *rebound*. The *impact* event, highlighted in the yellow solid line box, exhibits a sharp and powerful energy distribution with a higher magnitude in the lower frequencies than the *rebound*. The *rebound* marked with a blue dashed-line box shows a more prolonged and less intense energy distribution. Meanwhile, the *noise* in the red dots line boxes displays a more random and varied distribution across the frequency spectrum.

These events often occur in isolation and, unmistakably, sometimes these overlap, making recognition more laborious (Figure 6b). Distinguishing a ball sound from noise is challenging when background noise coincides with the shot. It can be difficult to separate the ball sound’s characteristics from competing noises, even in the spectrogram. Even in situations with high noise levels, the model should recognize and separate the ball sound, necessitating strong feature extraction and classification methods to consider overlapping sounds.

#### 2.3.2. Sound Classification Using Machine Learning

In order to classify the sounds from the tennis matches, several machine learning techniques (including Decision Tree, Random Forest, SVM, and XGBoost) were investigated during the development phase.

Two models were created to implement the classification of sound events. The first model was intended to distinguish between *impact* and sharp background *noise*, while the second model concentrated on identifying *rebounds* from *noise*. These models serve as the second step in our peak-identification process, ensuring that the recognized peaks in the audio data correspond to actual ball sounds (such as *impacts* or *rebounds*) rather than noises that may be interpreted as random or unexpected.

**Figure 7:**
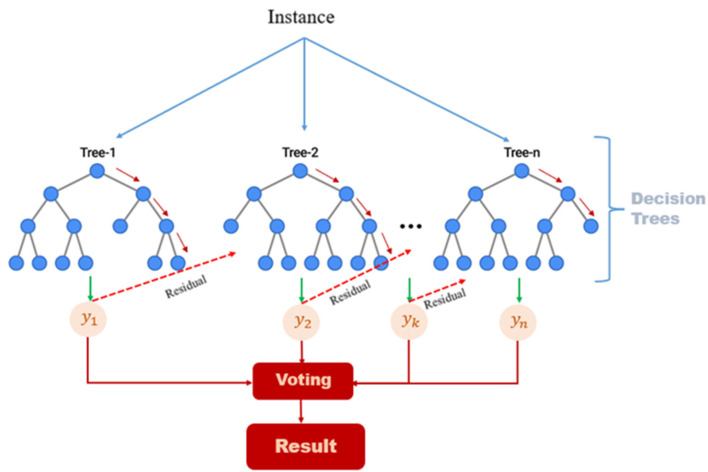
Illustration of the XGBoost Classifier: Tree-Based Learning and Voting Mechanism combining predictions from multiple decision trees through weighted voting.

For the first model, the XGBoost, a robust gradient-boosting algorithm known for its high performance on structured data (Figure 7) [53,54,55], consistently provided the highest accuracy with respect to the others and was employed. Indeed, its use only (binary classification, utilizing a learning rate of 0.1, 300 trees, and a maximum depth of 3) worked properly to differentiate between *noise* and *impacts*. This model performed adequately because *impact* sounds are sharp and distinct, making them easier to distinguish from background noise using the initial set of spectral and time-domain features.

The second model proved more difficult to implement because it had to identify the less harsh *rebound*s. Unlike the sharp sound of an *impact*, the *rebound*’s low amplitude made it difficult for the XGBoost model to separate it from noise consistently. To address this problem, we expanded our strategy by creating an ensemble of models, significantly increasing accuracy. The ensemble is a soft voting classifier that combines predictions from different models.

We were able to make use of each model’s advantages by combining XGBoost (configured for binary classification with a learning rate of 0.05, 500 trees, and five as a max depth) with a Support Vector Machine (SVM) (RBF kernel with C = 1.0, gamma = ‘scale’) [56] and a Multi-Layer Perceptron (MLP) [57] (single hidden layer with 100 neurons, using the Adam optimizer and ReLU activation) in a soft voting ensemble [58,59,60]. While SVM and MLP helped capture the subtle patterns needed to capture the *rebound* noises, XGBoost handled the more prominent aspects. A better classification performance was achieved with the soft voting method, which averages the projected probabilities of each model, especially for the problematic *rebound*-*noise* classification [61,62]. This ensemble approach provided a more robust solution and enhanced the overall accuracy.

#### 2.3.3. Model Training

Using the previously mentioned extracted features, we trained the classifier. The model was trained to recognize patterns and distinguish between the *impact* and *rebound* classes based on their unique acoustic signatures.

XGBoost is particularly well suited for this task due to its ability to handle a variety of features and its effectiveness in managing complex relationships in the data [57,58]. The model will be trained on a labeled dataset where each audio segment has been tagged as *impact*, *rebound*, or noise. To optimize performance, we used cross-validation and grid search [63,64] to fine-tune the model’s hyperparameters, including the learning rate, max depth, and number of estimators.

#### 2.3.4. Cross-Validation to Avoid Overfitting

**Figure 8:**
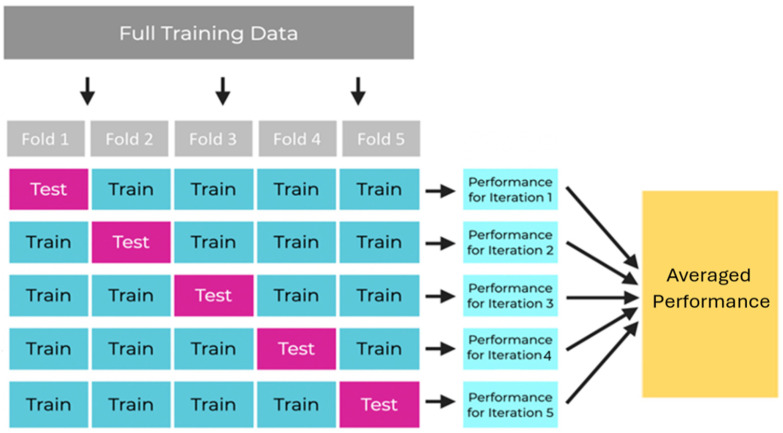
K-fFold cross-validation: data splitting and iterative testing process.

Cross-validation is one method for resolving the different model evaluation and selection issues [65,66,67]. In this project, we used k-fold cross-validation, which involves splitting the data into training and validation sets in specific ways: each fold containing 1120 samples for training and 280 for testing. The data are divided into five folds because, in our case, this provides a good balance between computational efficiency and robust evaluation. The process described below is shown in Figure 8:

1. Split the data into precisely five folds.
2. Train the model in four of the five folds (i.e., train the model in all the folds except one).
3. Evaluate the model on the 5th remaining fold by computing the accuracy.
4. Rotate the folds and repeat steps 2 and 3 with a new holdout fold. Repeat steps 2 and 3 until all k folds have been used as the holdout fold exactly 1 time.
5. Average the model performances across all iterations.

### 2.4. System Accuracy

In order to evaluate the accuracy of the system, the success of the results obtained was compared by video analysis conducted with BIOMOVIE—Ergo (Version 5.5) [68]. The video-analysis procedure allows the operator to identify the impacts and rebounds manually. The shooting mode was 1920 × 1080 px with a sampling rate of 240 fps. Consequently, the time absolute error of the camera can be assumed as equal to a frame time (*εt* = 4 ms) with a negligible jitter considering that the inaccuracy of the internal clock oscillator of the camera can be estimated at less than 0.1 μs. The repeatability was assessed by repeating the analysis multiple times and with a second operator.

### 2.5. Statistical Analysis

The Shapiro–Wilk test was used to validate the assumption of normality. Although the data followed a normal distribution, nonparametric tests were used for greater robustness because the sample was small. The Mann–Whitney test, Spearman’s correlation (ρ), and the percentage coefficient of variation for repeated measurements (CV%) were calculated to determine the system reliability for the rally rhythm and timing measurements obtained by the machine learning acoustic system and video analysis. Cohen’s weighted kappa (k) [69] and the standard error for k (SE) were used to assess the agreement for the rally rhythm and timing measurements. In addition, to determine the concordance between the two systems in the groundstrokes, intraclass correlation (ICC_3,1_) [70], 95% Confidence interval (CI) for ICC, and the Bland–Altman plot were used. Descriptive statistics and the Shapiro–Wilk test were used to assess the data distribution for the technical analysis. A statistical analysis was performed using Jasp software (Version 0.18.3) [71].

## 3. Results

### 3.1. Machine Learning Processing

#### 3.1.1. First Model Results

**Figure 9:**
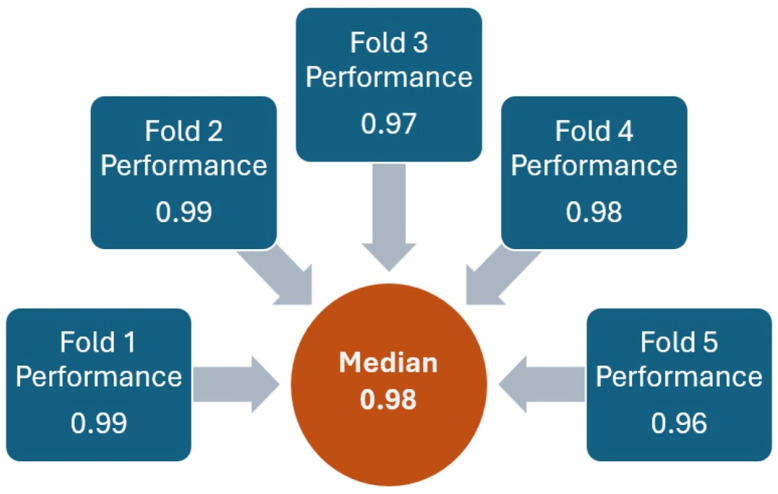
XGBoost model performance with five-fold cross-validation.

XGBoost was properly performed to distinguish between noise and *impact*. The model was trained, and the hyperparameters were optimized using random search. In the five-fold cross-validation, the accuracy across each fold ranged between 0.96 and 0.99, with a median value of 0.98 (Figure 9).

#### 3.1.2. Second Model Results

**Figure 10:**
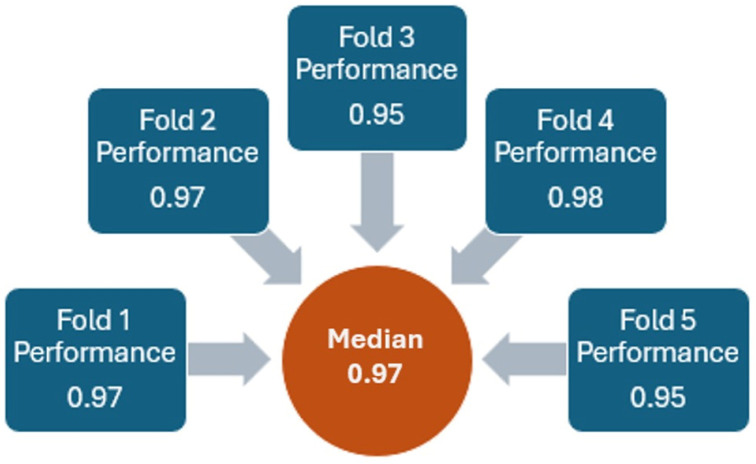
Ensemble model performance with five-fold cross-validation.

The ensemble model also performs properly by differentiating between the *rebound* and noise. The model was trained, and the hyperparameters were fine-tuned using random search. With a five-fold cross-validation, the accuracy for each fold ranged from 0.95 to 0.98, resulting in a median value of 0.97 (Figure 10). This consistency highlights the model’s effectiveness in accurately classifying the *rebound* sound despite background noise.

While the XGBoost model handled most of the classification, the two additional models enhanced the overall accuracy by capturing subtle patterns that improved the model’s performance, especially for complex cases when the *rebound* is shallow.

### 3.2. Video Analysis Repeatability

The repeatability results of video analysis showed good accuracy in retrieving time instants between the two operators (ICC = 0.98) and among repeated analyses by the same operator (ICC = 0.99).

### 3.3. Rally Assessment

In the rally assessment, in both the first and second cases, the system detected the *impact* of the examined player with an accuracy close to 100%. The rally rhythms assessed by the acoustic system presented no significant difference compared with the video analysis. The coefficient of variation for repeated measurements was also near zero (CV% = 0.19%). Cohen’s weighted kappa was equal to 0.99 (SE = 0.01).

**Figure 11:**
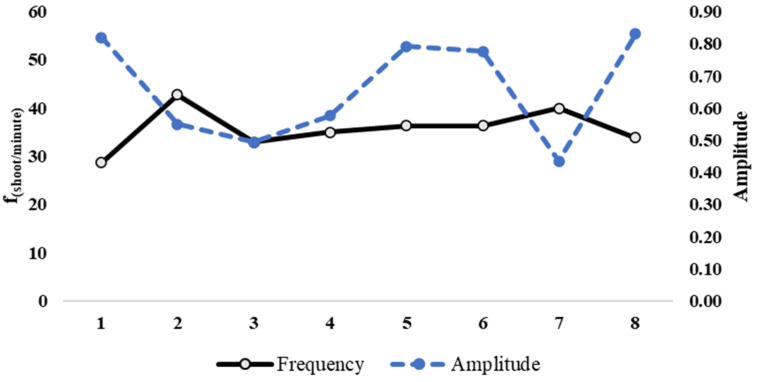
Assessment of *impact* sound amplitude (normalized positive peak) and instantaneous shot frequency (shoot/minute) in Rally 1. The solid line indicates the frequency, and the dashed line indicates the amplitude.

**Figure 12:**
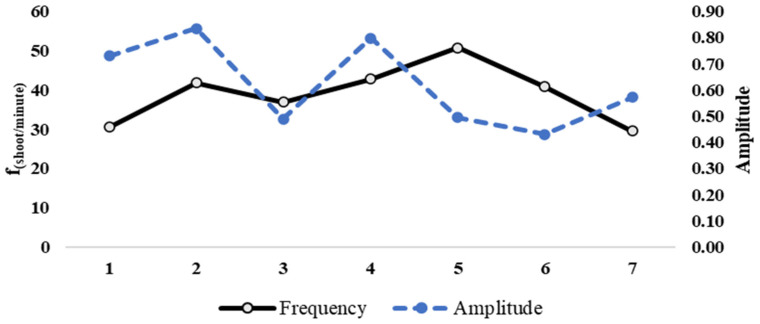
Assessment of *impact* sound amplitude (normalized positive peak) and instantaneous shot frequency (shoot/minute) in Rally 2. The solid line indicates the frequency, and the dashed line indicates the amplitude.

In Rally 1, the proposed system detected eight hits by the examined player (fifteen total hits played by the two players) in 23.03 s, with an average frequency of 39.08 shots per minute. In Rally 2, the system detected seven hits by the examined player (thirteen total hits played by the two players) in 18.29 s, with an average frequency of 42.65 shots per minute. Figure 11 and Figure 12 show the trend of the amplitude and frequency of hits during the first and second rallies, respectively.

### 3.4. Groundstrokes Assessment

In G1, 98 peaks with *impact* shape, 262 with *rebound* features, and 99.640 other sounds of different natures have been found. In G2, 85 peaks with *impact* shape, 130 with *rebound* features, and 99.785 sounds classified as noise have been found.

Machine learning processing validated these peaks, and among the 98 ones attributable to an *impact* in G1, 81 have been recognized as such, and 17 as noise. In addition, among the 262 peaks ascribable to a *rebound*, 70 have been identified as such, and the remaining 192 are classified as noise. As for G2, the ML algorithm recognized 57 *impacts* and 62 *rebounds*.

#### 3.4.1. System Accuracy

**Figure 13:**
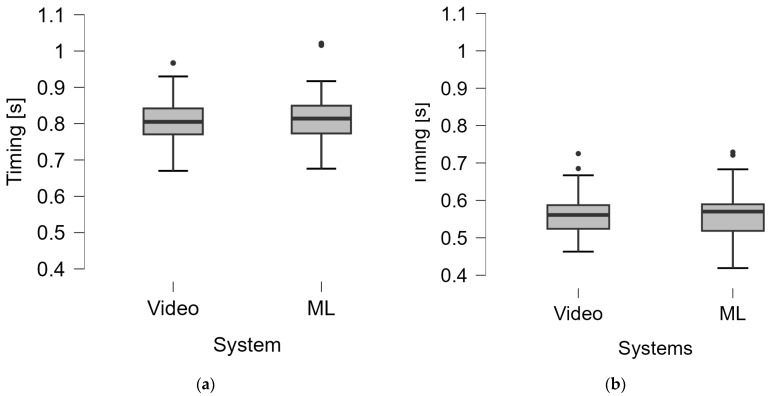
Timing assessment by the two systems in G1 (**a**) and G2 (**b**). Video, video analysis; ML, machine learning. (In both cases, the difference between the two groups was not significant according to the Mann–Whitney test).

In the timing assessment of groundstrokes, the comparison with video analysis showed that the system correctly recognized 78 *impacts* and 69 *rebounds* in G1, 48 *impacts*, and 40 *rebounds* in G2. The system correctly detected *impacts* in 96.25% of cases in G1 and 81.25% in G2 and *rebounds* in 86.25% of cases in G1 and 70.83% in G2. From the measurements taken by the two systems, the executive timing of the shots was calculated from which there were no statistically significant differences in the group medians shown in Figure 13 and Table 1 (G1 Δ = 9 ms, *p* = 0.415; G2 Δ = 9 ms, *p* = 0.943).

Spearman’s correlation between the measures of the two systems was strong in both groups: G1 ρ = 0.88 *p* < 0.001; G2 ρ = 0.90 *p* < 0.001 (Table 1). In the same table, the coefficient of variation (CV%), intraclass correlation (ICC**_3,1_**), 95% Confidence interval for ICC, Cohen’s weighted kappa (k), and k standard error (SE) for timing measurements of the two groups obtained by video analysis and the machine learning acoustic system are reported.

**Figure 14:**
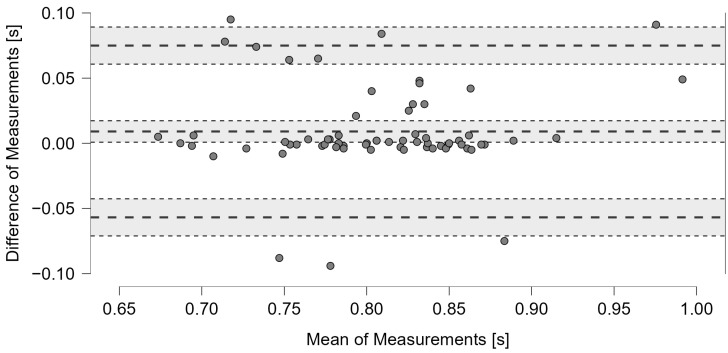
The Bland–Altman plot compares the timing values measured in G1 with the machine-learning acoustic system and video analysis. The graph features dashed bold lines indicating the mean and the limits of agreement of ±1.96 SD, while dotted lines indicate the 95% CIs.

In G1, the Bland–Altman showed agreement between the two methods, with an average difference of 0.009 s and a 95% CI between 0 and 0.017 s. Most points were within the limits of agreement of ±1.96 SD, with a slight tendency for overestimation by the proposed system (Figure 14).

**Figure 15:**
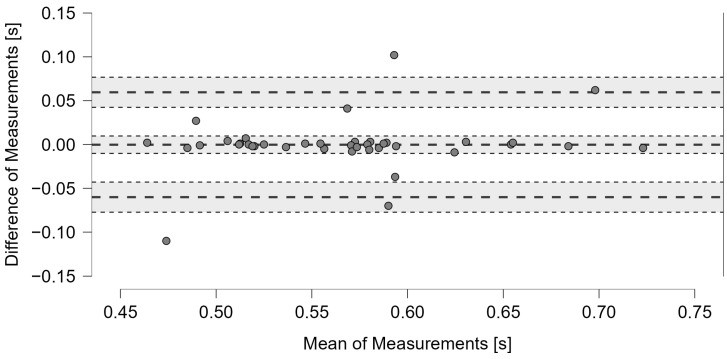
Bland–Altman plot comparing timing values measured in G2 by the machine learning acoustic system and video analysis. The graph features dashed bold lines indicating the mean and the limits of agreement of ±1.96 SD, while dotted lines indicate the 95% CIs.

In G2, the Bland–Altman results showed an average difference close to zero between the two methods and a value of 95% CI between −0.01 and 0.01 s. Almost all the points were within the limits of agreement of ±1.96 SD, indicating a strong correlation between the two methods (Figure 15).

#### 3.4.2. Technical Assessment

**Figure 16:**
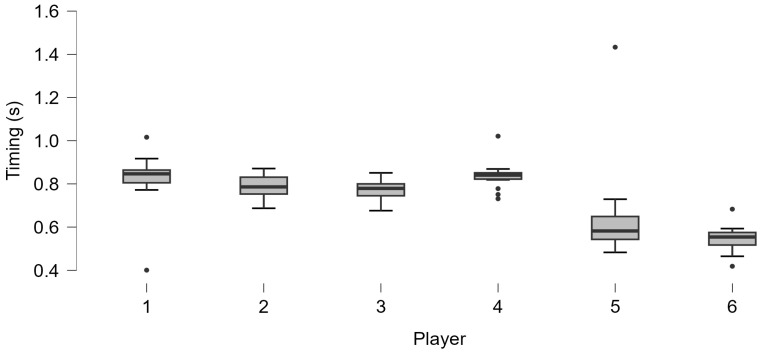
Technical timing assessment of the six players.

The preliminary on-court application of the system highlighted different executive timings on the six players of the two groups (G1 and G2) shown in Figure 16 and Table 2, in which the number of hits analyzed by the system (valid and misses), median and interquartile range (IQR), years of player experience, and significance levels of the Shapiro–Wilk test indicative of a non-normal distribution are reported.

## 4. Discussion

The current study explored the reliability of an acoustic event detection system in analyzing some technical–tactical skills of tennis players, such as rally rhythm and groundstroke timing [16,19].

The proposed system exploits ML techniques, already widespread in the literature [72,73], to classify specific acoustic events in tennis. Such techniques have recently become interesting in the sports field. In particular, a similar system has been proposed recently to estimate the height of vertical jumps by measuring the time of flight by identifying feet’ takeoff and landing sounds [74,75]. Other articles on recognizing typical tennis events [38,39,40,41,42], identified in the literature and cited above, have used similar acoustic-based techniques, although with different purposes, obtaining results comparable to those described in the present paper. In particular, some of these studies aimed at segmenting game phases and breaks to support match analysis systems and the automatic generation of tennis highlights [40,42]. Further studies have explored techniques for recognizing impact, rebound, and direction of spin played in the shot, although focused on a post-game offline analysis [41].

Therefore, to date, most papers available in the literature that exploit a similar acoustic approach are oriented to support match analysis tasks, which, as usual, are conducted after the matches have occurred. Conversely, this study is focused on validating an ML-based acoustic detection algorithm suited for the assessment of the temporal components of the game to provide the coach with immediate feedback on the court that can be useful for evaluating and training players.

For the analysis of the pace of the rally, simple use of peak detection and filtering procedures was adequate to ensure the proper detection of all events, as confirmed by the video analysis. On the contrary, in the case of the measurement of the executive timing of groundstrokes, the detection of *rebounds* was more difficult due to the presence of environmental noise at a level comparable with the *rebound* sound. Therefore, an ML classification procedure was necessary for differentiating between noise and the distinct *impact* or *rebound*.

The ML algorithm has been trained using a dataset containing 1400 audio samples that had previously been recorded. Several ML techniques, including Decision Tree, SVM, and others, were evaluated during the algorithm development phase. However, XGBoost [53,54,55] consistently provided the highest accuracy for this task. The features used for this classification were carefully selected from both time-domain and frequency-domain characteristics of the audio data. Moreover, we introduced an ensemble approach to enhance the model’s accuracy in distinguishing between noise and rebounds. For the first model (*impact* detection), XGBoost alone delivered high accuracy, allowing us to accomplish the task without including results from additional models.

On the other hand, for the second model (distinguishing the low-amplitude sound of the ball rebound on the ground from noise), we determined that a combination of three algorithms was necessary to achieve the desired level of accuracy in line with what is in the literature. For this reason, additional features (tonnetz, spectral contrast, and chroma) were used to improve the performance. The ensemble approach uses a soft voting classifier that combines predictions from XGBoost, an SVM [56], and an MLP [57]. The system’s effectiveness was evaluated through a k-fold cross-validation method [65,66,67]. A five-fold cross-validation provided a good balance between computational efficiency and robust evaluation. With k = 5, each fold contained 1120 samples for training and 280 for testing, which was a large enough sample size for effective learning and reliable assessment. Both the first and second models demonstrated high performance, achieving a median value of accuracy of 0.98 and 0.97, respectively. In addition, consistent accuracy across folds is a good indicator of model stability and the absence of overfitting.

### 4.1. System Accuracy

In the assessment of rally rhythm, the system shows high accuracy, compared with video analysis, in both hard and clay surfaces and with adjacent court sounds. This result is possible because the system relies on detecting the player’s *impact* close to the microphone. This sound is distinguishable by the system in frequency and amplitude from other sounds in the environment. In addition to the average rally rhythm, the system provides the instantaneous frequency of shots played, a handy parameter in structuring specific on-court exercises, for example, on those relative to rhythm variations.

As for the analysis of executive timing, the peaks detection function is less selective because the sounds to be screened are of different amplitudes and types (i.e., *impacts* and *rebounds*). The *rebound* sound level often is below the ambient noise level. For this reason, validating these sounds through a more performant technique like a machine learning algorithm is crucial to solving this task. In both situations analyzed, ambient noises were present: wind, voices, and road traffic in G1, as well as sounds coming from other courts in G2. The system has been proven to detect 96.25% and 81.25% of *impacts* in the two cases while correctly detecting 86.25% and 70.83% *rebounds*. This results in the executive timing detection of 109 out of 128 hits (69/80 in G1 and 40/48 in G2). Although the timing detection of some shots was lost, high measurement accuracy was found in those detected by the system compared with video analysis. No significant differences emerged between the executive timing recorded through the two systems, video analysis and audio (machine learning-based), and a strong correlation index, ICC, and Cohen’s weighted kappa were found in both G1 and G2. The coefficient of variation for repeated measures was modest (3.09% and 3.90%). These results show that ambient noises did not compromise accuracy, resulting in the correct measurement of executive timing of 86.3% of shots in G1 (69/80). However, in G2, sounds from the adjacent courts (e.g., *impacts* and *rebounds*) worsened the detection accuracy, resulting in fewer analyzed shots (83.3%), although it was in an environment free of wind and traffic noise.

### 4.2. Groundstrokes Technical Assessment

An example of a possible on-court application of the system (which is not intended to be a statistical investigation) was conducted on six amateur players who participated in the study with diverse experience and play levels, showing evidence of their different executive timing. Different timing distributions can be observed between each player: those with more years of experience have shorter executive timing, which is in line with what has been reported in the literature [43]. In addition, the players with more experience (greater than 2 years) presented less variability and a normal distribution of the executive timing, unlike the players with less playing experience [76]. In a controlled situation such as the one proposed, players with good experience master the technical gesture more than novices still in technical acquisition, attributable to the high number of repetitions resulting in an automation of the sports gesture [14]. This feature could be a helpful parameter for understanding the stroke technique’s acquisition stage. In addition, proper timing (neither too short nor too long) can allow for impact on the ball at an appropriate distance from the body and in an optimal spot, promoting less strain on the muscle–tendon and joint structures and helping reduce the risk of injury [28,29]. This feature and the real-time assessment of rally rhythm can be used to structure technical exercises in the court or tactical drills, such as rally rhythm variation exercises.

The results presented prompt us to consider the potential of this system in helpful on-court applications, both as a tool for evaluating players’ technical–tactical skills in closed and open situations and as a training aid.

### 4.3. Limitations of the Study

Although the system was shown to monitor some quantitative parameters in the game (i.e., rally rhythm and executive timing), the quality of the stroke, let alone its power and accuracy, was not analyzed in this study. At this juncture, accurate analyses are needed to assess how the type and wear condition of balls, racquets, and string tension affect the sound produced at impact, and the numerosity of these variables constitutes the main limitation of this system development.

Since this study aimed only to develop and test an acoustic event detection system in tennis without intending to conduct a large statistical investigation, the proposed on-court application example on a small sample of players cannot represent a survey of the game’s characteristics. In addition, a standardized investigation protocol was employed to analyze a single structured situation, thus limiting the extension of the results to other contexts and possible game situations that may occur during a game. The study of how using more microphones or their different positioning in the court may affect measurement quality has not been investigated. Furthermore, since the fundamental action of the toss in the serve is not detectable by an acoustic system, timing in this gesture was not assessed in this study.

### 4.4. Future Perspectives

The system proposed in this study, used in two-player rallies (singles tennis) and a structured technical drill, can be adopted in future work in other specific conditions. For example, the method shown in Section 2.1.1 (Rally Assessment) can be employed without distinction in singles or doubles. The same can be said for the groundstrokes timing assessment, which could be used in other tactical contexts (attack, defense, return, etc.) and the backhand stroke. Given its versatility, it can also be adapted to all racquet sports and, if preemptively set up to recognize double bounces before impact, could also find use in Wheelchair Tennis. Beyond these possible applications, the system could be refined in future work to investigate the stroke quality of all the fundamentals, including serve [77].

## 5. Conclusions

The results of the present study showed high accuracy levels for the presented system. Using a single simple microphone placed in a non-invasive position and a notebook made it possible to detect the rally rhythm and groundstroke timing of the assessed players. Although the system missed some shots in the timing assessment, those detected presented high accuracy in timing measurement compared with video analysis. Larger values and more significant variability were observed in the technical timing analysis in players with less playing experience, confirming the findings in the literature. These findings prompt us to consider the possible application of this system to provide real-time feedback in technical learning, helping the player to automate the gesture.

Overall, the proposed system can be a valuable, fast, and automatic assessment tool for investigating the technical–tactical skills of tennis players, helping to perfect these skills, improving game performance, and reducing injury risks. However, subsequent studies are needed to investigate all game situations, microphone configurations, and possible environmental situations and expand the examined subjects by involving athletes of different playing levels. Future studies could be implemented to improve the algorithm’s efficiency and study qualitative aspects of the shot, such as effect, power, and accuracy at the *impact* point.

## Figures and Tables

**Table 1:** Timing assessment in the two groups (G1 and G2): ML, Machine learning; n, number of correct events detected; IQR, interquartile range; ρ, Spearman’s correlation coefficient; CV%, coefficient of variation % for repeated measurements; ICC, intraclass correlation coefficient; 95% CI, 95% Confidence interval for ICC; k, Cohen’s weighted kappa; SE, standard error for k.

Groups	Detection System	Video		ML							
		n	Timing (s) Median; IQR	n	Timing (s) Median; IQR	ρ	CV% ^a^	ICC_3,1_	95% CI	k	SE
G1	*Rebound* detection	80	0.81; 0.07	69	0.81; 0.08	0.88 ***	3.09	0.88	0.83 to 0.91	0.872	0.038
	*Impact* detection	80		78							
G2	*Rebound* detection	48	0.56; 0.06	40	0.57; 0.07	0.90 ***	3.90	0.87	0.82 to 0.91	0.904	0.046
	*Impact* detection	48		48							

^a^ Root mean square method; *** *p* < 0.001.

**Table 2:** Technical timing assessment of the six players: N, number of shoots assessed; SD, Standard Deviation; experience (Years), years of playing experience of each player.

Player	N (Valid; Missing)	Median; IQR	Experience (Years)	*p*-Value Shapiro–Wilk
#1	18; 2	0.85; 0.06	1	<0.001 *
#2	17; 3	0.79; 0.08	2.5	0.848
#3	17; 3	0.78; 0.06	2.5	0.339
#4	17; 4	0.84; 0.03	1	0.004 *
#5	19; 5	0.58; 0.11	1.5	<0.001 *
#6	21; 3	0.55; 0.06	5	0.275

* *p*-values less than 0.05 equals a data distribution that does not follow the normal one.

## Data Availability

The raw data supporting the conclusions of this article will be made available by the corresponding author upon request.

## References

[1] Casale L. (2003). Physical Training for Tennis Players.

[2] Caprioli L., Campoli F., Edriss S., Padua E., Panichi E., Romagnoli C., Annino G., Bonaiuto V. Video Analysis Application to Assess the Reaction Time in an ATP Tennis Tournament. Proceedings of the 11th International Conference on Sport Sciences Research and Technology Support.

[3] Issurin V.B. (2010). New Horizons for the Methodology and Physiology of Training Periodization. Sports Med..

[4] Matveev L.P. (2001). Teoría General Del Entrenamiento Deportivo.

[5] Fox E.L., Bowers R.W., Foss M.L. (1993). The Physiological Basis for Exercise and Sport.

[6] Schönborn R. (1999). Advanced Techniques for Competitive Tennis.

[7] Sogut M., Ayser S., Akar G.B. (2017). A Video-Based Analysis of Rhythmic Accuracy and Maintenance in Junior Tennis Players. Pamukkale J. Sport Sci..

[8] Aleksovski A. (2015). Precision of ground strokes in tennis. Act. Phys. Educ. Sport.

[9] Elliott B. (2006). Biomechanics and Tennis. Br. J. Sports Med..

[10] Kibler W.B., Sciascia A. (2004). Kinetic Chain Contributions to Elbow Function and Dysfunction in Sports. Clin. Sports Med..

[11] Roetert E.P., Kovacs M. (2019). Tennis Anatomy.

[12] Sciascia A., Thigpen C., Namdari S., Baldwin K. (2012). Kinetic Chain Abnormalities in the Athletic Shoulder. Sports Med. Arthrosc. Rev..

[13] Faneker E., van Trigt B., Hoekstra A. (2021). The Kinetic Chain and Serve Performance in Elite Tennis Players.

[14] Verrelli C.M., Caprioli L., Bonaiuto V. (2024). Cyclic Human Movements and Time-Harmonic Structures: Role of the Golden Ratio in the Tennis Forehand. Proceedings of the International Workshop on Engineering Methodologies for Medicine and Sport.

[15] Zachopoulou E., Mantis K. (2001). The Role of Rhythmic Ability on the Forehand Performance in Tennis. Eur. J. Phys. Educ..

[16] Smekal G., Von Duvillard S.P., Rihacek C., Pokan R., Hofmann P., Baron R., Tschan H., Bachl N. (2001). A Physiological Profile of Tennis Match Play. Med. Sci. Sports Exerc..

[17] Filipcic A., Zecic M., Reid M., Crespo M., Panjan A., Nejc S. (2015). Differences in Performance Indicators of Elite Tennis Players in the Period 1991–2010. J. Phys. Educ. Sport.

[18] Reid M., McMurtrie D., Crespo M. (2010). The Relationship between Match Statistics and Top 100 Ranking in Professional Men’s Tennis. Int. J. Perform. Anal. Sport.

[19] Castellani A., D’Aprile A., Tamorri S. (2007). Tennis Training.

[20] Guevara D. Tap Tempo Button 2022. https://play.google.com/store/apps/details?id=com.diegoguevara.taptempo.

[21] Cureton T.K., Coe D.E. (1933). An Analysis of the Errors in Stop-Watch Timing. Res. Q. Am. Phys. Educ. Assoc..

[22] Faux D.A., Godolphin J. (2019). Manual Timing in Physics Experiments: Error and Uncertainty. Am. J. Phys..

[23] Amir N., Saifuddin (2018). Analysis of Body Position, Angle and Force in Lawn Tennis Service Accuracy. J. Phys. Educ. Sport.

[24] Kang Y.-T., Lee K.-S., Seo K.-W. (2006). Analysis of Lower Limb Joint Angle and Rotation Angle of Tennis Forehand Stroke by Stance Pattern. Korean J. Sport Biomech..

[25] Mark King A.H., Blenkinsop G. (2017). The Effect of Ball Impact Location on Racket and Forearm Joint Angle Changes for One-Handed Tennis Backhand Groundstrokes. J. Sports Sci..

[26] Caprioli L., Romagnoli C., Campoli F., Edriss S., Padua E., Bonaiuto V., Annino G. (2025). Reliability of an Inertial Measurement System Applied to the Technical Assessment of Forehand and Serve in Amateur Tennis Players. Bioengineering.

[27] Takahashi H., Wada T., Maeda A., Kodama M., Nishizono H. (2009). An Analysis of Time Factors in Elite Male Tennis Players Using the Computerised Scorebook for Tennis. Int. J. Perform. Anal. Sport.

[28] Reid M., Elliott B., Crespo M. (2013). Mechanics and Learning Practices Associated with the Tennis Forehand: A Review. J. Sports Sci. Med..

[29] Fu M.C., Ellenbecker T.S., Renstrom P.A., Windler G.S., Dines D.M. (2018). Epidemiology of Injuries in Tennis Players. Curr. Rev. Musculoskelet. Med..

[30] Hernández-Belmonte A., Sánchez-Pay A. (2021). Concurrent Validity, Inter-Unit Reliability and Biological Variability of a Low-Cost Pocket Radar for Ball Velocity Measurement in Soccer and Tennis. J. Sports Sci..

[31] Bortolotti C., Coviello A., Giannattasio R., Lambranzi C., Serrani A., Villa G., Xu H., Aliverti A., Esmailbeigi H. (2023). Wearable Platform for Tennis Players Performance Monitoring: A Proof of Concept. Proceedings of the 2023 IEEE International Workshop on Sport, Technology and Research (STAR).

[32] Brito A.V., Fonseca P., Costa M.J., Cardoso R., Santos C.C., Fernandez-Fernandez J., Fernandes R.J. (2024). The Influence of Kinematics on Tennis Serve Speed: An In-Depth Analysis Using Xsens MVN Biomech Link Technology. Bioengineering.

[33] Kelley J., Choppin S., Goodwill S., Haake S. (2010). Validation of a Live, Automatic Ball Velocity and Spin Rate Finder in Tennis. Procedia Eng..

[34] Tian B., Zhang D., Zhang C. (2020). High-Speed Tiny Tennis Ball Detection Based on Deep Convolutional Neural Networks. Proceedings of the 2020 IEEE 14th International Conference on Anti-counterfeiting, Security, and Identification (ASID).

[35] Qazi T., Mukherjee P., Srivastava S., Lall B., Chauhan N.R. (2015). Automated Ball Tracking in Tennis Videos. Proceedings of the 2015 Third International Conference on Image Information Processing (ICIIP).

[36] Annino G., Bonaiuto V., Campoli F., Caprioli L., Edriss S., Padua E., Panichi E., Romagnoli C., Romagnoli N., Zanela A. (2023). Assessing Sports Performances Using an Artificial Intelligence-Driven System. Proceedings of the 2023 IEEE International Workshop on Sport, Technology and Research (STAR).

[37] Edriss S., Romagnoli C., Caprioli L., Zanela A., Panichi E., Campoli F., Padua E., Annino G., Bonaiuto V. (2024). The Role of Emergent Technologies in the Dynamic and Kinematic Assessment of Human Movement in Sport and Clinical Applications. Appl. Sci..

[38] Martin C., Kulpa R., Ropars M., Delamarche P., Bideau B. (2013). Identification of Temporal Pathomechanical Factors during the Tennis Serve. Med. Sci. Sports Exerc..

[39] Zhang B., Dou W., Chen L. (2006). Ball Hit Detection in Table Tennis Games Based on Audio Analysis. Proceedings of the 18th International Conference on Pattern Recognition (ICPR’06).

[40] Baughman A., Morales E., Reiss G., Greco N., Hammer S., Wang S. Detection of Tennis Events from Acoustic Data. Proceedings of the the 2nd International Workshop on Multimedia Content Analysis in Sports.

[41] Yamamoto N., Nishida K., Itoyama K., Nakadai K. Detection of Ball Spin Direction Using Hitting Sound in Tennis. Proceedings of the 8th International Conference on Sport Sciences Research and Technology Support (ICSPORTS).

[42] Huang Q., Cox S., Zhou X., Xie L. (2012). Detection of Ball Hits in a Tennis Game Using Audio and Visual Information. Proceedings of the 2012 Asia Pacific Signal and Information Processing Association Annual Summit and Conference.

[43] Caprioli L., Campoli F., Edriss S., Frontuto C., Najlaoui A., Padua E., Romagnoli C., Annino G., Bonaiuto V. (2024). Assessment of Tennis Timing Using an Acoustic Detection System.

[44] Multimedia Programming Interface and Data Specifications 1.0 1991. https://www.mmsp.ece.mcgill.ca/Documents/AudioFormats/WAVE/Docs/riffmci.pdf.

[45] Shouran M., Elgamli E. (2020). Design and Implementation of Butterworth Filter. Int. J. Innov. Res. Sci. Eng. Technol..

[46] Hussin S.F., Birasamy G., Hamid Z. (2016). Design of Butterworth Band-Pass Filter. Politek. Kolej Komuniti J. Eng. Technol..

[47] Breebaart J., McKinney M.F. (2004). Features for Audio Classification. Algorithms in Ambient Intelligence.

[48] Zhang T., Kuo C.-C.J. (2001). Audio Content Analysis for Online Audiovisual Data Segmentation and Classification. IEEE Trans. Speech Audio Process..

[49] Rong F. (2016). Audio Classification Method Based on Machine Learning. Proceedings of the 2016 International Conference on Intelligent Transportation, Big Data & Smart City (ICITBS).

[50] Anitta D., Joy A., Ramaraj K., Thilagaraj M. (2023). Implementation of Machine Learning and Audio Visualizer to Understand the Emotion of a Patient. Proceedings of the 2023 International Conference on Sustainable Computing and Smart Systems (ICSCSS).

[51] Lo P.-C., Liu C.-Y., Chou T.-H. (2022). DNN Audio Classification Based on Extracted Spectral Attributes. Proceedings of the 2022 14th International Conference on Signal Processing Systems (ICSPS).

[52] Logan B. (2000). Others Mel Frequency Cepstral Coefficients for Music Modeling.

[53] Chen T., Guestrin C. Xgboost: A Scalable Tree Boosting System. Proceedings of the 22nd ACM SIGKDD International Conference on Knowledge Discovery and Data Mining.

[54] Liu Y., Yin Y., Zhu Q., Cui W. (2022). Musical Instrument Recognition by XGBoost Combining Feature Fusion. arXiv.

[55] Gusain R., Sonker S., Rai S.K., Arora A., Nagarajan S. (2022). Comparison of Neural Networks and XGBoost Algorithm for Music Genre Classification. Proceedings of the 2022 2nd International Conference on Intelligent Technologies (CONIT).

[56] Hearst M.A., Dumais S.T., Osuna E., Platt J., Scholkopf B. (1998). Support Vector Machines. IEEE Intell. Syst. Their Appl..

[57] Popescu M.-C., Balas V.E., Perescu-Popescu L., Mastorakis N. (2009). Multilayer Perceptron and Neural Networks. WSEAS Trans. Circuits Syst..

[58] Awe O.O., Opateye G.O., Johnson C.A.G., Tayo O.T., Dias R. (2024). Weighted Hard and Soft Voting Ensemble Machine Learning Classifiers: Application to Anaemia Diagnosis. Sustainable Statistical and Data Science Methods and Practices: Reports from LISA 2020 Global Network, Ghana, 2022.

[59] Abro A.A., Talpur M.S.H., Jumani A.K., Sıddıque W.A., Yaşar E. (2021). Voting Combinations-Based Ensemble: A Hybrid Approach. Celal Bayar Univ. J. Sci..

[60] Pavlyshenko B. (2018). Using Stacking Approaches for Machine Learning Models. Proceedings of the 2018 IEEE Second International Conference on Data Stream Mining & Processing (DSMP).

[61] Ansari M.R., Tumpa S.A., Raya J.A.F., Murshed M.N. (2021). Comparison between Support Vector Machine and Random Forest for Audio Classification. Proceedings of the 2021 International Conference on Electronics, Communications and Information Technology (ICECIT).

[62] Prasanna D.L., Tripathi S.L. (2022). Machine Learning Classifiers for Speech Detection. Proceedings of the 2022 IEEE VLSI Device Circuit and System (VLSI DCS).

[63] Liashchynskyi P., Liashchynskyi P. (2019). Grid Search, Random Search, Genetic Algorithm: A Big Comparison for NAS. arXiv.

[64] Belete D.M., Huchaiah M.D. (2022). Grid Search in Hyperparameter Optimization of Machine Learning Models for Prediction of HIV/AIDS Test Results. Int. J. Comput. Appl..

[65] Berrar D. (2019). Others Cross-Validation. https://dberrar.github.io/papers/Berrar_EBCB_2nd_edition_Cross-validation_preprint.pdf.

[66] Yates L.A., Aandahl Z., Richards S.A., Brook B.W. (2023). Cross Validation for Model Selection: A Review with Examples from Ecology. Ecol. Monogr..

[67] Bates S., Hastie T., Tibshirani R. (2024). Cross-Validation: What Does It Estimate and How Well Does It Do It?. J. Am. Stat. Assoc..

[68] Villanis P. (2024). BIOMOVIE Ergo Software, Version 5.5.

[69] Cohen J. (2013). Statistical Power Analysis for the Behavioral Sciences.

[70] Shrout P.E., Fleiss J.L. (1979). Intraclass Correlations: Uses in Assessing Rater Reliability. Psychol. Bull..

[71] Wagenmakers E.-J. (2024). Jasp Software.

[72] Tang G., Liang R., Xie Y., Bao Y., Wang S. (2019). Improved Convolutional Neural Networks for Acoustic Event Classification. Multimed. Tools Appl..

[73] Kong Q., Xu Y., Sobieraj I., Wang W., Plumbley M.D. (2019). Sound Event Detection and Time–Frequency Segmentation from Weakly Labelled Data. IEEE/ACM Trans. Audio Speech Lang. Process..

[74] Pueo B., Lopez J.J., Jimenez-Olmedo J.M. (2019). Audio-Based System for Automatic Measurement of Jump Height in Sports Science. Sensors.

[75] Banchero L., Lopez J.J., Pueo B., Jimenez-Olmedo J.M. (2024). Combining Sound and Deep Neural Networks for the Measurement of Jump Height in Sports Science. Sensors.

[76] Cowin J., Nimphius S., Fell J., Culhane P., Schmidt M. (2022). A Proposed Framework to Describe Movement Variability within Sporting Tasks: A Scoping Review. Sports Med.-Open.

[77] Paté A., Petel M., Belhassen N., Chadefaux D. (2024). Radiated Sound and Transmitted Vibration Following the Ball/Racket Impact of a Tennis Serve. Vibration.

